# Prevalence of Hallux Valgus in Patients With Femoral Neck Fracture and Its Association With Postoperative Functional Outcomes

**DOI:** 10.1002/jfa2.70226

**Published:** 2026-10-07

**Authors:** Kazuki Kanazawa, Shunsuke Akiho, Masahiko Sakai, Ken Ichikawa, Soshiro Goshima, Ryohei Haraguchi, Takuaki Yamamoto

**Affiliations:** ^1^ Orthopaedic Surgery Fukuoka Seisyukai Hospital Kasuya‐machi Fukuoka Japan; ^2^ Department of Orthopaedic Surgery Fukuoka University Faculty of Medicine Jonan‐ku Fukuoka Japan

**Keywords:** femoral neck fracture, functional independence measure, hallux valgus, older adults, postoperative function

## Abstract

**Background:**

Hallux valgus is a frequent forefoot deformity in older adults and has been linked to impaired balance, gait dysfunction, and increased fall risk. However, its prevalence among patients with femoral neck fracture and its association with postoperative functional outcomes remain unclear.

**Methods:**

This single‐center retrospective study included 235 patients aged ≥ 65 years with available radiographic data who underwent surgery for femoral neck fracture between 2022 and 2024. Hallux valgus was defined on weight‐bearing radiographs as a hallux valgus angle (HVA) ≥ 20°. Functional outcomes were evaluated using the Functional Independence Measure (FIM) at 3 months postoperatively. Multivariable linear regression analysis was performed to determine whether hallux valgus was independently associated with motor FIM scores after adjustment for age, sex, surgical procedure, and cognitive function.

**Results:**

Among the 235 patients with radiographic data, 178 (76%) were women, with a median age of 85 (77–90) years; 86% of patients underwent bipolar hemiarthroplasty. Hallux valgus was present in 55% (130/235), including 20% (47/235) with moderate‐to‐severe deformity (HVA ≥ 30°). Patients with hallux valgus had significantly lower motor FIM scores at 3 months than those without the deformity (*p* = 0.044), whereas cognitive and total FIM scores did not differ. In multivariable analysis, hallux valgus (HVA ≥ 20°) was independently associated with lower motor FIM scores (*B* = −5.92, 95% CI −10.95 to −0.89; *p* = 0.021).

**Conclusions:**

Hallux valgus is highly prevalent in patients with femoral neck fracture and is independently associated with lower postoperative motor function. Assessment of hallux valgus may provide additional insight into postoperative functional prognosis in this population.

## 1 Introduction

Foot deformities have been associated with impaired balance, gait dysfunction, and an increased risk of falls in older adults [1, 2]. Hallux valgus, one of the most common foot deformities in older adults, may contribute to these impairments through foot pain, altered foot alignment, and reduced hallux function [2].

Hallux valgus has been reported to reduce contact between the hallux and the ground, leading to decreased load‐bearing function of the forefoot [3]. This may result in instability during standing and impaired propulsion during gait, thereby contributing to impaired balance [4, 5]. Indeed, hallux valgus has been associated with an increased risk of falls in older adults [6]. Such impairment of foot function may adversely affect postural stability and walking ability. In more severe cases, hallux valgus may also be accompanied by pain and functional limitation, which may increase the likelihood of falls and potentially contribute to fracture risk [7].

Hip fracture is a major public health concern in aging societies, often leading to substantial declines in activities of daily living and an increased need for long‐term care [8]. Several risk factors for femoral neck fracture, a common type of hip fracture, have been identified, including demographic characteristics, osteoporosis, comorbidities, previous fractures, and poor nutritional status [9].

Because falls are a major cause of femoral neck fractures, foot morphology may influence fracture risk. Furthermore, because hallux valgus is associated with impaired gait and balance, it may also be relevant to functional outcomes after fracture. However, the prevalence of hallux valgus among patients with femoral neck fracture and its association with postoperative motor function remain unclear.

Therefore, the aim of this study was to investigate the prevalence and severity of hallux valgus in patients with femoral neck fracture. In addition, we examined its association with postoperative motor function at 3 months.

## 2 Materials and Methods

### 2.1 Ethical Approval

This study was conducted in accordance with the Declaration of Helsinki and was approved by the institutional ethics committee of our hospital (approval number: 2022‐06).

A study information document was provided to all eligible patients, and the study was explained during the perioperative consultation. Informed consent for participation in this study was obtained from all patients.

### 2.2 Patient Characteristics

Consecutive patients aged ≥ 65 years who underwent surgery for femoral neck fractures at our hospital between January 2022 and December 2024 were included in this study. Femoral neck fractures were selected because surgical procedures and postoperative rehabilitation are relatively uniform compared with intertrochanteric fractures, allowing early postoperative weight bearing.

The following cases were excluded:

- Patients with foot amputation.
- Patients with a history of foot surgery.
- Pathological fractures due to metastatic bone tumors or metabolic bone diseases (e.g., osteomalacia).
- Rheumatoid foot or Charcot foot.
- Age < 65 years.

Patients younger than 65 years were excluded because treatment strategies and postoperative rehabilitation protocols differed from those used for older adults with fragility femoral neck fractures.

In patients who sustained sequential fractures during the study period, the second fracture was excluded to avoid duplication, and only the first fracture was included in the analysis. Ultimately, 240 patients were included in the study. The study flow is shown in Figure 1.

**FIGURE 1:**
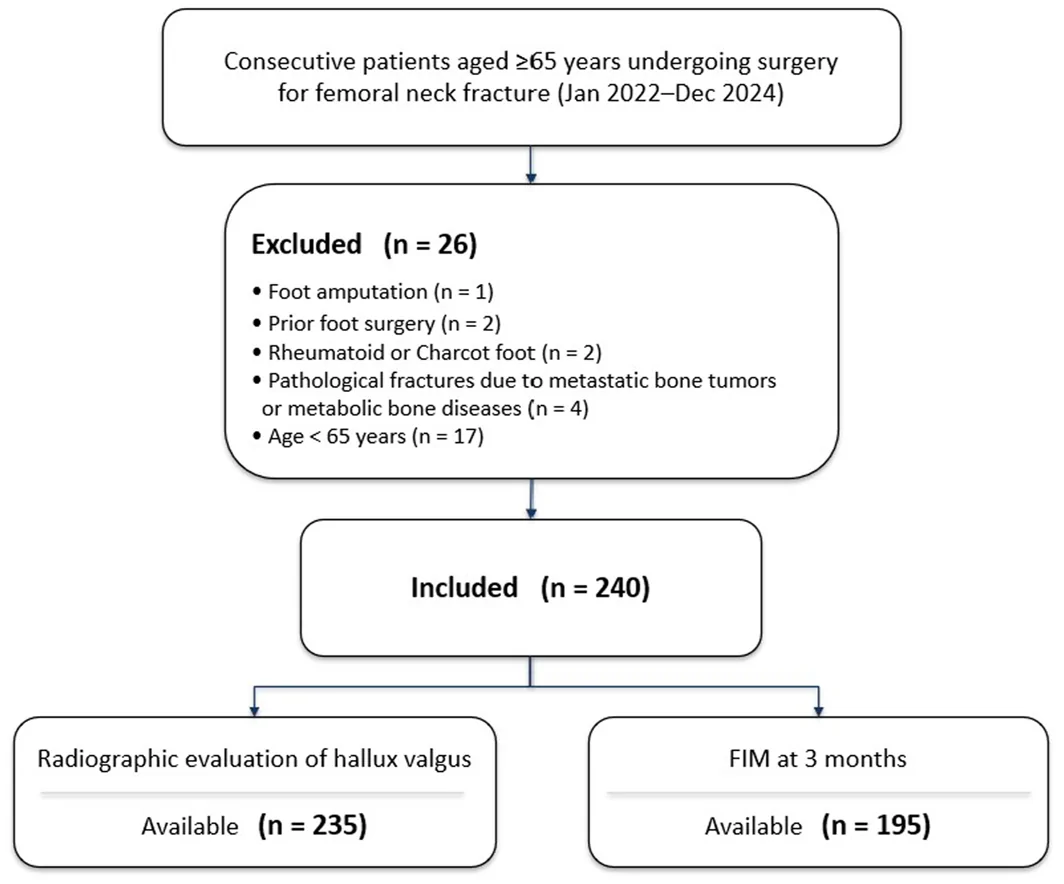
Study flow diagram.

### 2.3 Radiographic Evaluations

To evaluate foot morphological characteristics, weight‐bearing anteroposterior radiographs of both feet were obtained 1 week postoperatively. When full standing weight‐bearing was feasible, radiographs were obtained in the standing position. When standing was not feasible, radiographs were obtained in a seated position with as much forefoot loading as tolerated. The hallux valgus angle (HVA), first–second intermetatarsal angle (M1M2A), and first–fifth intermetatarsal angle (M1M5A) were measured (Figure 2). Hallux valgus was defined as an HVA of ≥ 20°. Severity was categorized as mild (20°–29°), moderate (30°–39°), and severe (≥ 40°) according to the criteria proposed by Coughlin [10].

**FIGURE 2:**
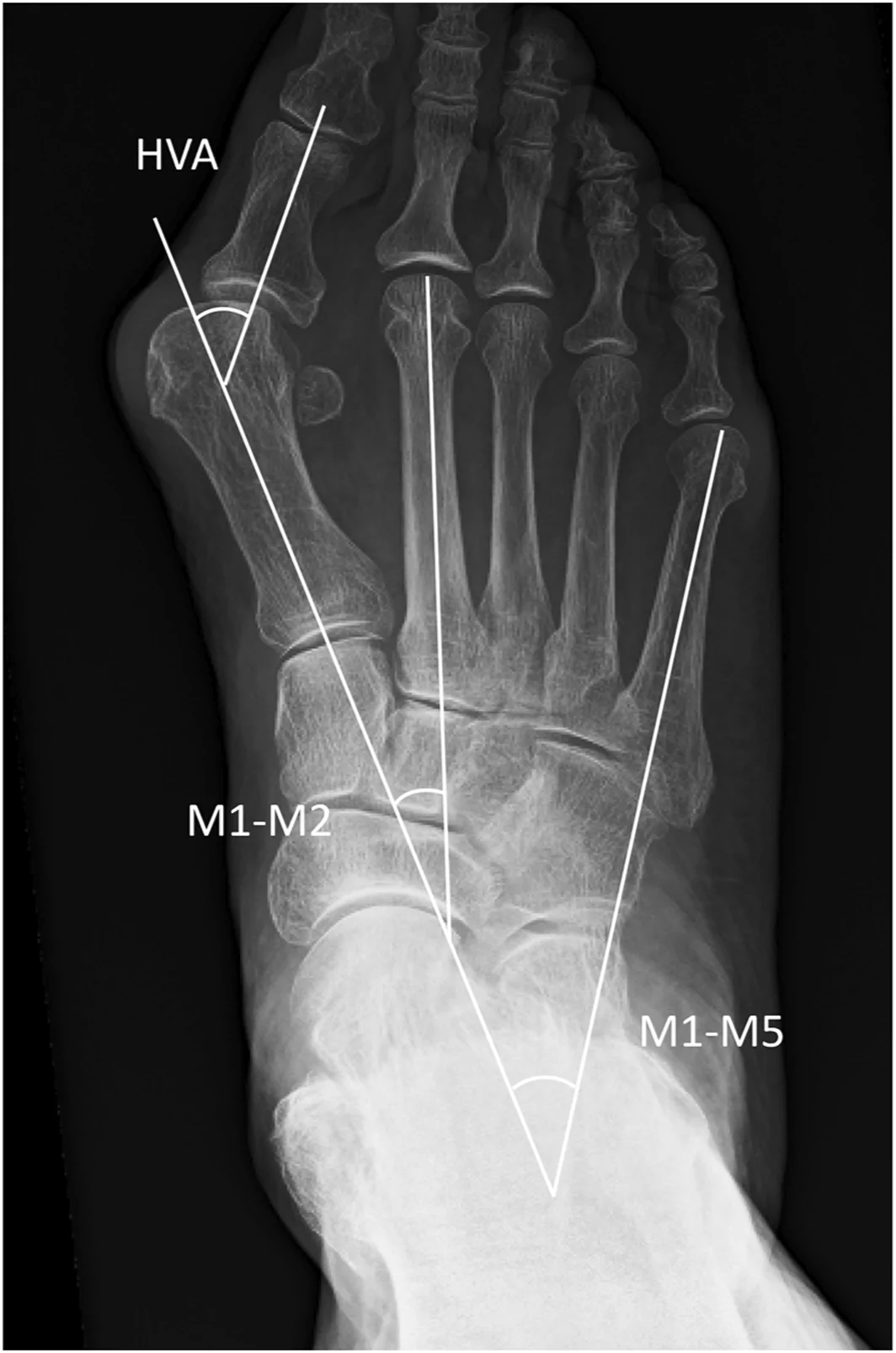
Radiographic measurements of hallux valgus angle (HVA), first–second intermetatarsal angle (M1–M2), and first–fifth intermetatarsal angle (M1–M5).

Femoral neck fractures were evaluated using anteroposterior radiographs of both hips according to the Garden classification [11]. In this study, Garden stages I and II were defined as nondisplaced fractures, whereas Garden stages III and IV were defined as displaced fractures. At our institution, open reduction and internal fixation (ORIF) was generally performed for nondisplaced fractures, whereas bipolar hemiarthroplasty (BHA) was performed for displaced fractures.

Angle measurements were independently performed by two board‐certified orthopedic surgeons. Reproducibility was assessed using intraclass correlation coefficients (ICCs). The intra‐observer ICC was 0.90 and the inter‐observer ICC was 0.89, indicating excellent agreement.

### 2.4 Functional Evaluations

Functional status was evaluated using the Functional Independence Measure (FIM) at 3 months postoperatively. The FIM is a validated instrument widely used in rehabilitation settings to assess the level of independence in activities of daily living [12, 13]. It consists of a motor domain (motor FIM; 13 items, score range 13–91) and a cognitive domain (cognitive FIM; 5 items, score range 5–35), with a total score ranging from 18 to 126. Higher scores indicate greater functional independence. FIM assessments were performed by experienced physical therapists trained in the standardized FIM assessment procedure.

Cognitive function was evaluated using the Revised Hasegawa Dementia Scale (HDS‐R) [14]. The HDS‐R consists of nine items assessing orientation, memory, attention, and verbal fluency, with scores ranging from 0 to 30. Higher scores indicate better cognitive function. A cutoff score of 20 is commonly used for screening cognitive impairment.

### 2.5 Clinical and Laboratory Assessments

Blood test results obtained at admission were used for analysis. Nutritional status was assessed using the Geriatric Nutritional Risk Index (GNRI), which was calculated based on serum albumin level and body weight [15].

Bone mineral density (BMD) was measured using dual‐energy X‐ray absorptiometry (DXA; GE Lunar iDXA, GE Healthcare). Lumbar spine and proximal femoral BMD were measured on postoperative day 7. The presence of osteoporosis treatment was confirmed at admission. Patients were defined as receiving treatment if they were using active vitamin D analogs, bisphosphonates, selective estrogen receptor modulators (SERMs), denosumab (anti‐RANKL antibody), or teriparatide.

### 2.6 Patient Flow and Rehabilitation Protocol

Of the 240 patients included in the study, radiographic evaluation of hallux valgus was possible in 235. Five patients were excluded from this analysis because three were transferred to another hospital within one week after surgery, and two were unable to undergo weight‐bearing radiography because of poor general condition. Of the 235 patients eligible for functional assessment, FIM scores at 3 months postoperatively were available for 195 patients (83.0%). Missing data (*n* = 40) were due to death within 3 months after surgery (*n* = 5), reoperation due to postoperative complications (*n* = 2), and loss to follow‐up (*n* = 33) (Figure 1).

Postoperative rehabilitation was initiated on the first postoperative day in all patients. Weight bearing was also started from the first postoperative day under the supervision of physical therapists. Although the progression of rehabilitation was adjusted according to each patient's condition, the overall rehabilitation protocol remained broadly standardized.

### 2.7 Statistical Analysis

Hallux valgus was assessed in both feet, and the larger HVA was used to represent each patient. Continuous variables were assessed for normality using the Shapiro–Wilk test. Because several variables showed non‐normal distributions, between‐group comparisons were performed using the Mann–Whitney U test. Categorical variables were compared using the chi‐square test. Multivariable linear regression analysis was performed using the FIM motor score after 3 months as the dependent variable. Age, sex, surgical procedure, HDS‐R score, and HVA ≥ 20° were included as independent variables based on clinical relevance. Linear regression assumptions were satisfied, and variance inflation factors ranged from 1.0 to 2.0, indicating no multicollinearity. Statistical significance was set at *p* < 0.05. Analyses were performed using SPSS (version 29.0; IBM Corp., Armonk, NY, United States).

Analyses were performed using available data: prevalence analysis included patients with radiographic data (*n* = 235), and multivariable analysis included those with complete 3‐month FIM data (*n* = 195). Given the retrospective design, potential selection bias and residual confounding cannot be excluded.

During the preparation of this manuscript, ChatGPT (OpenAI) was used for language editing and refinement. The authors take full responsibility for the content.

## 3 Results

### 3.1 Patient Characteristics

A total of 235 patients with available radiographic data were included. Fifty‐seven were men and 178 were women (75.7%). The median age was 85 years (IQR, 77–90). Median height, body weight, and body mass index (BMI) were 152 (147–158) cm, 45 (39–53) kg, and 19.5 (17.2–21.8) kg/m^2^, respectively.

For radiographic assessment, the maximum hallux valgus angle (HVA) between the right and left feet was used. The median HVA was 20 (15–26)°, the median first–second intermetatarsal angle (M1–M2) was 11 (9–14)°, and the median first–fifth intermetatarsal angle (M1–M5) was 28 (25–31)°. Regarding surgical procedures, 32 patients (13.6%) underwent open reduction and internal fixation (ORIF), and 203 patients (86.4%) underwent bipolar hemiarthroplasty (BHA).

At 3 months postoperatively, the median motor FIM score was 57 (37–84), the median cognitive FIM score was 25 (13–34), and the median total FIM score was 82 (50–117). In addition, the median cognitive function score assessed by the revised Hasegawa Dementia Scale (HDS‐R) was 16 (6–26), the median serum albumin level was 3.8 (3.4–4.1) g/dL, and the median Geriatric Nutritional Risk Index (GNRI) was 94 (86–101). The baseline patient characteristics are summarized in Table 1.

**TABLE 1:** Characteristics of the study participants (*n* = 235).

	Total (*n* = 235)	Men (*n* = 57)	Women (*n* = 178)
Demographic characteristics			
Age (yr), median (IQR)	85 (77–90)	80 (73–88)	86 (78–91)
Height (cm)	152 (147–158)	164 (159–170)	150 (145–153)
Weight (kg)	45 (39–53)	53 (46–57)	43 (38–49)
Body mass index (kg/m^2^)	19.5 (17.2–21.8)	19.5 (17.2–21.3)	19.5 (17.3–21.8)
Radiographic evaluations			
Hallux valgus angle (HVA, °)	20 (15–26)	18 (15–22)	21 (15–28)
First–second intermetatarsal angle (M1–M2, °)	11 (9–14)	10 (8–12)	12 (10–15)
First–fifth intermetatarsal angle (M1–M5, °)	28 (25–31)	27 (23–29)	29 (26–32)
Surgical procedures			
ORIF, *n* (%)	32 (13.6%)	10 (17.5%)	22 (12.4%)
BHA, *n* (%)	203 (86.4%)	47 (82.5%)	156 (87.6%)
Functional evaluations			
FIM motor score	57 (37–84)	63 (40–83)	57 (37–84)
FIM cognitive score	25 (13–34)	28 (19–33)	23 (13–35)
FIM total score	82 (50–117)	84 (57–116)	78 (49–118)
Bone health evaluations			
Lumbar spine BMD (g/cm^2^)	0.907 (0.776–1.058)	1.071 (0.908–1.173)	0.872 (0.752–0.992)
Total hip BMD (g/cm^2^)^a^	0.621 (0.552–0.700)	0.699 (0.631–0.776)	0.602 (0.528–0.669)
Osteoporosis medication, *n* (%)	47 (20.0%)	2 (3.5%)	45 (25.3%)
Systemic assessments			
Serum albumin (g/dL)	3.8 (3.4–4.1)	3.7 (3.3–4.1)	3.8 (3.5–4.2)
GNRI	94 (86–101)	92 (86–100)	95 (87–101)
HDS‐R score	16 (6–26)	18 (10–26)	15 (5–26)

Abbreviations: BHA, bipolar hemiarthroplasty; BMD, bone mineral density; FIM, Functional Independence Measure; GNRI, Geriatric Nutritional Risk Index; HDS‐R, Hasegawa Dementia Scale‐Revised; ORIF, open reduction and internal fixation.

^a^ Only patients with unilateral fractures were included.

### 3.2 Prevalence of Hallux Valgus

The prevalence of hallux valgus among patients with femoral neck fractures is shown in Figure 3. Hallux valgus (HVA ≥ 20°) was observed in 130 patients (55.3%). Of these, moderate hallux valgus (30° ≤ HVA < 40°) was present in 34 patients (14.5%), and severe hallux valgus (HVA ≥ 40°) in 13 patients (5.5%).

**FIGURE 3:**
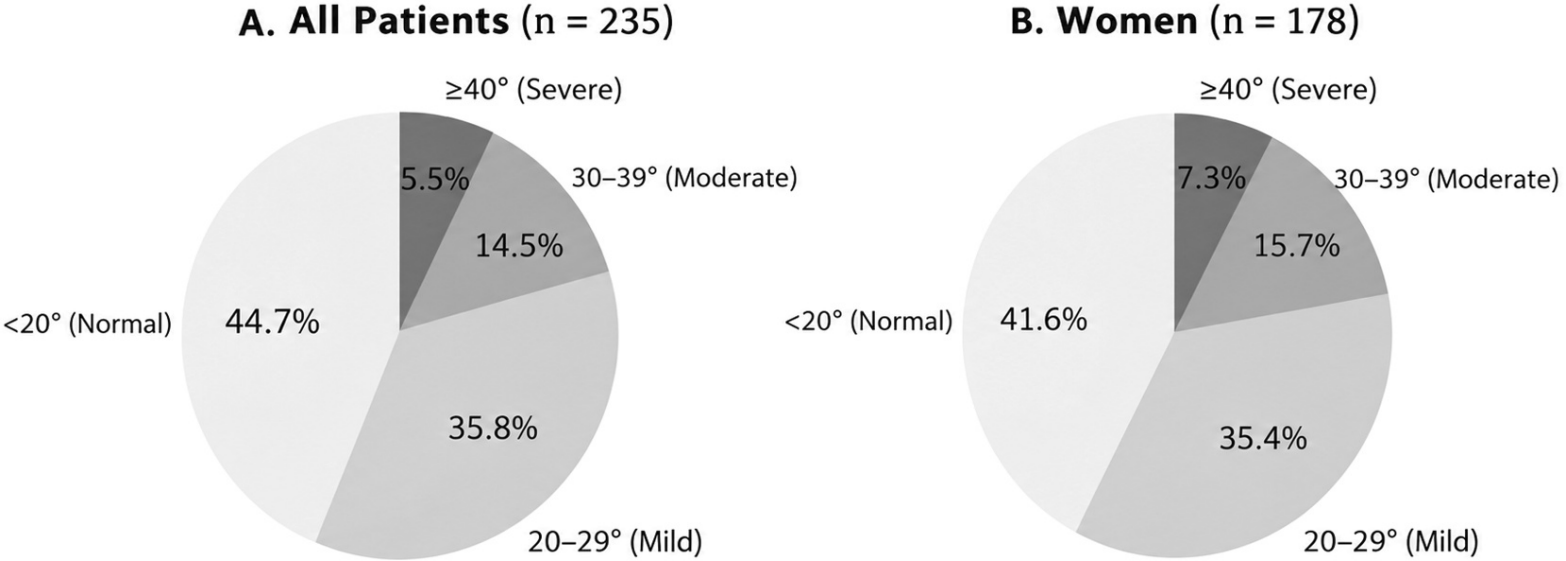
Distribution of hallux valgus severity in all patients (A) and women (B).

Among female patients, hallux valgus was observed in 104 of 178 patients (58.4%). Moderate hallux valgus (30° ≤ HVA < 40°) was present in 28 patients (15.7%), and severe hallux valgus (HVA ≥ 40°) in 13 patients (7.3%).

### 3.3 Comparison Between Hallux Valgus and Normal Groups (HVA ≥ 20° Vs. < 20°)

To examine differences in patient characteristics and postoperative functional outcomes associated with hallux valgus, patients were divided into a hallux valgus group (HVA ≥ 20°, *n* = 130) and a normal group (HVA < 20°, *n* = 105). There were no significant differences between the groups in age, sex, body mass index, or surgical procedure. The hallux valgus group had significantly lower height and body weight than the normal group. The FIM motor score at 3 months was significantly lower in the hallux valgus group than in the normal group (*p* = 0.044). In contrast, no significant differences were observed in FIM cognitive or total scores. No significant differences were observed between the groups in lumbar or hip bone mineral density, nutritional status, or cognitive function (Table 2). In a supplementary analysis using a higher threshold (HVA ≥ 30°), patients with HVA ≥ 30° showed lower motor FIM scores; however, the difference was not statistically significant (*p* = 0.198) (Supporting Information S1: Table S1).

**TABLE 2:** Comparison between hallux valgus and normal groups (*n* = 235).

	Hallux valgus group (HVA ≥ 20°, *n* = 130)	Normal group (HVA < 20°, *n* = 105)	*p*‐value
Demographic characteristics			
Age (yr), median (IQR)	84 (77–89)	87 (78–91)	0.215
Sex (female), *n* (%)	104 (80.0%)	74 (70.5%)	0.123
Height (cm)	150 (145–157)	153 (148–160)	**0.006**
Weight (kg)	43.5 (38.8–50.0)	46.3 (40.0–54.3)	**0.013**
Body mass index (kg/m^2^)	19.1 (17.1–21.6)	19.7 (17.4–21.9)	0.215
Surgical procedures (ORIF), *n* (%)	16 (12.3%)	16 (15.2%)	0.646
Functional evaluations			
FIM motor score	51 (35–82)	66 (38–87)	**0.044**
FIM cognitive score	23 (13–34)	29 (14–34)	0.356
FIM total score	77 (49–115)	91 (53–121)	0.120
Bone health evaluations			
Lumbar spine BMD (g/cm^2^)	0.89 (0.76–1.04)	0.93 (0.81–1.08)	0.121
Total hip BMD (g/cm^2^)^a^	0.61 (0.53–0.69)	0.63 (0.57–0.71)	0.239
Osteoporosis medication, *n* (%)	22 (16.9%)	25 (24.0%)	0.236
Systemic assessments			
Serum albumin (g/dL)	3.8 (3.4–4.1)	3.9 (3.5–4.2)	0.236
GNRI	92 (85–99)	95 (88–102)	0.058
HDS‐R score	15 (6–26)	17 (8–26)	0.583

*Note:* Bold values indicate statistical significance (*p* < 0.05).

Abbreviations: BHA, bipolar hemiarthroplasty; BMD, bone mineral density; FIM, Functional Independence Measure; GNRI, Geriatric Nutritional Risk Index; HDS‐R, Hasegawa Dementia Scale‐Revised; ORIF, open reduction and internal fixation.

^a^ Only patients with unilateral fractures were included.

### 3.4 Multivariable Regression Analysis

In the multivariable linear regression analysis, age, sex, surgical procedure, HVA ≥ 20°, and cognitive function assessed by the HDS‐R were included as independent variables (Table 3). Each one‐year increase in age was associated with a decrease in FIM motor score (*B* = −0.54, 95% CI −0.87 to −0.20, *p* = 0.002). HVA ≥ 20° was independently associated with a lower FIM motor score (*B* = −5.92, 95% CI −10.95 to −0.89, *p* = 0.021). The HDS‐R score was also significantly associated with FIM motor score (*B* = 1.61, 95% CI 1.34 to 1.87, *p* < 0.001), whereas surgical procedure was not significantly associated. In an additional multivariable analysis including an interaction term between sex and hallux valgus, the interaction was not significant (Supporting Information S1: Table S2).

**TABLE 3:** Multivariable linear regression analysis of factors associated with FIM motor score at 3 months (*n* = 195).

	B	95% CI	*p*‐value
Age (yr)	−0.54	−0.87 to −0.20	**0.002**
Female sex	5.69	−0.35 to 11.73	0.065
ORIF (vs. BHA)	3.16	−3.88 to 10.20	0.377
Hallux valgus, HVA ≥ 20°	−5.92	−10.95 to −0.89	**0.021**
HDS‐R score	1.61	1.34 to 1.87	**< 0.001**

*Note:* Bold values indicate statistical significance (*p* < 0.05).

Abbreviations: BHA, bipolar hemiarthroplasty; HDS‐R, Hasegawa Dementia Scale‐Revised; HVA, hallux valgus angle; ORIF, open reduction and internal fixation.

## 4 Discussion

The most important finding of this study is the high prevalence of hallux valgus (55%) in patients with femoral neck fracture. In addition, hallux valgus was independently associated with lower postoperative motor FIM scores. The magnitude of the difference was relatively small and should therefore be interpreted with caution. These findings suggest that hallux valgus may be associated with postoperative functional outcomes in hip fractures.

### 4.1 Prevalence and Severity of Hallux Valgus

The prevalence of hallux valgus among patients with femoral neck fracture was 55.3%, and it was particularly common in women. In addition, moderate‐to‐severe deformity (HVA ≥ 30°) accounted for 20% of cases. These findings are consistent with epidemiological studies reporting that hallux valgus is more common in older women [16].

Previous studies in community‐dwelling older adults have reported prevalence rates of 41.1% using weight‐bearing radiographs and 35% using footprint analysis [17, 18]. In a large epidemiological study, Hannan et al. also reported a prevalence of approximately 40% among community‐dwelling older adults [19]. Although differences in assessment methods should be considered, the prevalence observed in the present study was higher than in these reports, suggesting that hallux valgus may be more prevalent among patients with femoral neck fractures. These patients are at a high risk of falls, and abnormal foot alignment may be one potential contributing factor.

### 4.2 Hallux Valgus and Postoperative Motor Function

In this study, patients with hallux valgus showed lower motor FIM scores at 3 months postoperatively than those without hallux valgus. In addition, no significant difference was observed in cognitive FIM, indicating that the impact of hallux valgus may primarily relate to lower‐limb function and mobility.

Hallux valgus has been reported to reduce the push‐off function of the hallux and cause abnormal foot alignment, which may affect gait efficiency and balance [20, 21]. These functions are directly related to postural stability and gait reacquisition during postoperative rehabilitation, and such impairments may adversely affect motor recovery. Previous studies have also reported associations between hallux valgus and impaired physical performance, balance function, and falls risk in older adults [1, 2]. These findings may support the association between hallux valgus and lower postoperative motor FIM scores observed in the present study. From a clinical perspective, assessment of hallux valgus may help identify patients who require particular attention to gait and balance during postoperative rehabilitation. Recognition of this foot deformity may therefore provide complementary information for evaluating functional prognosis after femoral neck fracture.

Hallux valgus remained independently associated with lower motor FIM scores after adjustment for age, sex, surgical procedure, and cognitive function. This finding suggests that the association between hallux valgus and postoperative motor function was not fully explained by age, sex, surgical procedure, or cognitive function.

In the supplementary analysis using a higher hallux valgus threshold (HVA ≥ 30°), lower motor FIM scores were also observed, although the difference was not statistically significant. In addition, no significant interaction was observed between sex and hallux valgus. Although foot‐specific assessments such as foot pain, plantar pressure distribution, balance function, and gait parameters were not evaluated, the observed association should be interpreted with caution when considering the underlying mechanisms.

## 5 Limitations

This study has several limitations. First, this was a single‐center retrospective study, which may limit the generalizability of the findings. Second, postoperative motor function was evaluated using the FIM at 3 months, and follow‐up data were unavailable for 40 of 235 patients (17.0%). Therefore, the findings should be interpreted with caution, although baseline characteristics were generally similar between patients with and without follow‐up data (Supporting Information S1: Table S3). In addition, pre‐fracture functional status was not assessed and may have influenced postoperative motor outcomes. Third, full weight‐bearing was not feasible in all patients at the time of radiographic assessment. Therefore, HVA may have been underestimated in some patients. Fourth, potential confounding factors, such as foot pain and postoperative rehabilitation intensity or adherence, were not available and could not be included in the analysis. Therefore, residual confounding may remain. In addition, foot‐specific outcome measures were not available in this retrospective cohort. Future studies incorporating foot‐specific outcome measures are needed to clarify the mechanisms underlying the association between hallux valgus and postoperative motor function.

## 6 Conclusion

Hallux valgus is highly prevalent in patients with femoral neck fracture and is independently associated with lower postoperative motor function. Assessment of hallux valgus may provide additional insight into postoperative functional prognosis in this population.

## Author Contributions

**Kazuki Kanazawa:** conceptualization, methodology, investigation. **Shunsuke Akiho:** writing – original draft, formal analysis, data curation, visualization. **Masahiko Sakai:** investigation, data curation. **Ken Ichikawa:** investigation, data curation. **Soshiro Goshima:** investigation. **Ryohei Haraguchi:** investigation. **Takuaki Yamamoto:** supervision, writing – review and editing.

## Funding

The authors have nothing to report.

## Ethics Statement

This study was approved by the Ethics Committee of Fukuoka Seisyukai Hospital (approval number: 2022‐06) and was conducted in accordance with the Declaration of Helsinki.

## Consent

The authors have nothing to report.

## Conflicts of Interest

The authors declare no conflicts of interest.

## Supporting information


Supporting Information S1

## Data Availability

The data that support the findings of this study are available on request from the corresponding author. The data are not publicly available due to privacy or ethical restrictions.
