# Supplementary material for: Prevalence of Hallux Valgus in Patients With Femoral Neck Fracture and Its Association With Postoperative Functional Outcomes
Source: J Foot Ankle Res. 2026 Oct 7;19(4):e70226. doi: 10.1002/jfa2.70226 (PMC13646485; doi:10.1002/jfa2.70226)
Supplement: Supplementary file 1 [file JFA2-19-e70226-s001.pdf]

Supplementary Table S1.

Comparison Between Moderate-to-Severe Hallux Valgus and Normal Groups (n=152)

	**Moderate-to-Severe Hallux valgus group (HVA ≥30°, n=47)**	**Normal group (HVA <20°, n=105)**	**P-value**
**Demographic characteristics**			
Age (yr)	85 (77-91)	87 (78-91)	0.801
Sex (Female), n (%)	42 (89.4%)	74 (70.5%)	**0.010**
Height (cm)	147 (144-156)	153 (148-160)	**< 0.001**
Weight (kg)	42 (36-46)	46.3 (40.0-54.3)	**0.001**
Body mass index (kg/m²)	18.4 (17.1-21.0)	19.7 (17.4-21.9)	0.117
Surgical procedures (ORIF), n (%)	6 (12.8%)	16 (15.2%)	0.790
**Functional evaluations**			
FIM motor score	50 (30-83)	66 (38-87)	0.198
FIM cognitive score	25 (15-35)	29 (14-34)	0.769
FIM total score	77 (48-118)	91 (53-121)	0.365
**Bone health evaluations**			
Lumbar spine BMD (g/cm²)	0.87 (0.75-1.07)	0.93 (0.81-1.08)	0.264
Total hip BMD (g/cm²)*	0.60 (0.53-0.68)	0.63 (0.57-0.71)	0.132
Osteoporosis medication, n (%)	8 (17.0%)	25 (24.0%)	0.842
**Systemic assessments**			
Serum albumin (g/dL)	3.7 (3.4-4.0)	3.9 (3.5-4.2)	0.131
GNRI	91 (83-99)	95 (88-102)	**0.038**
HDS-R score	19 (6-26)	17 (8-26)	0.773

* Only patients with unilateral fractures were included.

Abbreviations: ORIF, open reduction and internal fixation; FIM, Functional Independence Measure; BMD, bone mineral density; GNRI, Geriatric Nutritional Risk Index; HDS-R, Hasegawa Dementia Scale-Revised

In a supplementary analysis, patients with HVA ≥30° were compared with those with HVA <20°. No significant differences were observed in postoperative FIM motor, cognitive, or total scores between the groups. Although patients with HVA ≥30° tended to have lower FIM motor scores, the difference did not reach statistical significance.

Supplementary Table S2.

Multivariable linear regression analysis with an interaction term (Hallux valgus × sex)

	B	95% CI	P-value
Age (yr)	-0.54	-0.86 to -0.20	**0.002**
Female sex	6.02	-2.13 to 14.16	0.147
ORIF (vs BHA)	3.16	-3.90 to 10.22	0.378
Hallux valgus, HVA ≥20°	-5.37	-15.79 to 5.04	0.310
HDS-R score	1.60	1.34 to 1.87	**< 0.001**
Hallux valgus × sex	-0.72	-12.57 to 11.14	0.905

Abbreviations: ORIF, open reduction and internal fixation; BHA, bipolar hemiarthroplasty; HVA, hallux valgus angle; HDS-R, Hasegawa Dementia Scale-Revised

The interaction between hallux valgus and sex was not significant. The association between hallux valgus and postoperative motor function was therefore generally similar in men and women.

Supplementary Table S3.

Comparison Between Follow-up Available and Unavailable Groups (n = 235)

	**Follow-up available (n=195)**	**Follow-up unavailable (n=40)**	**P-value**
Age (yr)	84 (78-89)	87 (76-92)	0.295
Sex (Female), n (%)	152 (77.9%)	26 (65.0%)	0.082
Body mass index (kg/m²)	19.6 (17.2-21.6)	18.8 (16.4-22.3)	0.399
Surgical procedures (ORIF), n (%)	29 (14.9%)	3 (7.5%)	0.216
HVA (°)	20 (15-27)	20 (16-21)	0.061
HVA ≥20°, n (%)	108 (55.4%)	22 (55.0%)	0.964
HDS-R score	17 (6-26)	13 (7-23)	0.375

Abbreviations: ORIF, open reduction and internal fixation; HVA, hallux valgus angle

Baseline characteristics were compared between patients with and without available 3-month follow-up FIM data. No significant differences were observed in age, sex, body mass index, surgical procedure, hallux valgus severity, hallux valgus prevalence, or cognitive function. These findings suggest that loss to follow-up was unlikely to substantially bias the observed association between hallux valgus and postoperative motor function.
